# A case of ectopic pancreas of the stomach accompanied by intraductal papillary mucinous neoplasm with *GNAS* mutation

**DOI:** 10.1186/s12957-021-02424-x

**Published:** 2021-10-21

**Authors:** Naoko Nambu, Takashi Yamasaki, Nami Nakagomi, Tsutomu Kumamoto, Tatsuro Nakamura, Akio Tamura, Toshihiko Tomita, Hiroto Miwa, Hisashi Shinohara, Seiichi Hirota

**Affiliations:** 1 Department of Surgical Pathology, Hyogo College of Medicine, 1-1 Mukogawacho, Nishinomiya, Hyogo 663-8501 Japan; 2 Upper Gastrointestinal Division, Department of Surgery, Hyogo College of Medicine, 1-1 Mukogawacho, Nishinomiya, Hyogo 663-8501 Japan; 3 Division of Gastroenterology and Hepatology, Department of Internal Medicine, Hyogo College of Medicine, 1-1 Mukogawacho, Nishinomiya, Hyogo 663-8501 Japan

**Keywords:** Ectopic pancreas, Heterotopic pancreas, Pancreatic heterotopia, Intraductal papillary mucinous neoplasm, IPMN, *GNAS* mutation

## Abstract

**Background:**

Ectopic pancreas is basically a benign disease and is not always necessary to be removed. However, all types of neoplasms occurring in the normal pancreas such as ductal adenocarcinomas and intraductal papillary mucinous neoplasms (IPMNs) may develop even within ectopic pancreas. We recently encountered an extremely rare case of ectopic pancreas in the gastric antrum associated with IPMN possessing a GNAS mutation.

**Case presentation:**

A 71-year-old Japanese woman complained of epigastric pain. Computed tomography and upper gastrointestinal endoscopy showed an intramural cystic mass in the antrum of the stomach. Endoscopic ultrasound-guided fine needle aspiration (EUS-FNA) biopsy did not give a definitive diagnosis, and the patient underwent resection of the lesion. Histology of the resected specimen showed that the gastric intramural lesion was ectopic pancreas. Moreover, the lesion contained dilated duct components with tubulo-villous epithelial proliferation consistent with pancreatic IPMN. Since the covering epithelial cells had highly atypical nuclei, the lesion was diagnosed as IPMN with high grade dysplasia. Immunohistochemistry showed that the IPMN component showed to be MUC2-, MUC5AC-, and CDX2-positive but MUC1- and MUC6-negative. Mutational analyses using genomic DNA revealed that the IPMN component had a mutation of *GNAS* at exon 8 (Arg201Cys).

**Conclusion:**

We finally diagnosed this case as gastric ectopic pancreas accompanied by intestinal type IPMN with high grade dysplasia possessing *GNAS* mutation. Although there were 17 cases of ectopic pancreas with IPMN including 6 cases of gastric ones reported in the English literature, this is the first case of ectopic pancreas with IPMN which was proved to have *GNAS* mutation. Intimate preoperative examinations including imaging analyses and EUS-FNA biopsy/cytology are recommended to decide whether the lesion has to be resected or not even if they are not effective for getting the right diagnosis.

## Background

Ectopic pancreas, also known as pancreatic heterotopia or aberrant pancreas, is defined as pancreatic tissue that presents in organs other than the pancreas. Its predilection site is the stomach, duodenum, and Meckel’s diverticulum of the ileum, and it often affects submucosal, proper muscular and/or subserosal layers of them [1, 2]. Ectopic pancreas is considered to be a congenital and developmental abnormality in the process of embryogenesis. According to histological classification of ectopic pancreas by Heinrich, type I lesion has all 3 components of pancreatic tissue, namely ducts, acinar cells and Langerhans islets, type II has ducts and acinar cells, and type III has only ductal structure [1, 2]. Ectopic pancreas of the stomach is a rare condition occurring at a frequency of 0.9% in gastrectomy cases [1, 2]. On the other hand, it is relatively common among gastric submucosal masses which include gastrointestinal stromal tumor (GIST), leiomyoma, and schwannoma, and is usually found in the antrum [1, 2].

In the pancreas, many different types of neoplasms can occur. Most of them originate from pancreatic exocrine tissue especially duct component. Ductal adenocarcinomas are the most common tumor type of ductal origin with solid characterization, and intraductal papillary mucinous neoplasms (IPMNs) are a less common tumor type of ductal origin with cystic change. Although IPMNs generally proliferate within the pancreatic duct, they could have intraductal malignant lesion with rather high incidence and even might invade outside the ductal structure as the result of tumor progression. Thus, they can be divided into three categories: intraductal papillary mucinous adenoma (IPMA), IPMN with high-grade dysplasia (so-called non-invasive intraductal papillary mucinous carcinoma, IPMC), and invasive IPMC.

On the other hand, IPMNs can be classified into four subtypes by histology and immunohistochemistry (IHC) for mucin core proteins (MUCs)/CDX2: gastric, intestinal, pancreatobiliary, and oncocytic types [3, 4]. Gastric type generally shows immunohistochemical characteristics of MUC1(−)/MUC2(−)/MUC5AC(+)/MUC6(−)/CDX2(−), intestinal type MUC1(−)/MUC2(+)/MUC5AC(+)/MUC6(−)/CDX2(+), pancreatobiliary, and oncocytic types MUC1(+)/MUC2(−)/MUC5AC(+)/MUC6(+)/CDX2(−) [3, 4]. Pancreatobiliary and oncocytic types are distinguished by the presence of oncocytic change on histology.

Pancreatic neoplasms such as pancreatic duct adenocarcinomas, IPMNs and solid pseudopapillary neoplasms might have gene mutations of *K-ras*, *GNAS*, *p16*, *TP53*, *CTNNB1*, etc [5, 6]. Among these mutations, *K-ras* mutation could be found in both pancreatic duct adenocarcinomas and IPMNs, but *GNAS* mutation at exon 8 (Arg201Cys or Arg201His) is specifically detected in IPMNs [5, 6].

Ectopic pancreas is basically a benign disease and does not always require resection. However, all types of neoplasms occurring in the normal pancreas such as ductal adenocarcinomas and IPMNs may develop even within ectopic pancreas [6–21]. Therefore, ectopic pancreas might need to be surgically resected depending on the situation. We recently encountered an extremely rare case of ectopic pancreas (Heinrich type I) in the gastric antrum accompanied by intestinal type IPMN with high grade dysplasia possessing *GNAS* mutation. We report here the case with a review of the literature.

## Case presentation

**Fig. 1:**
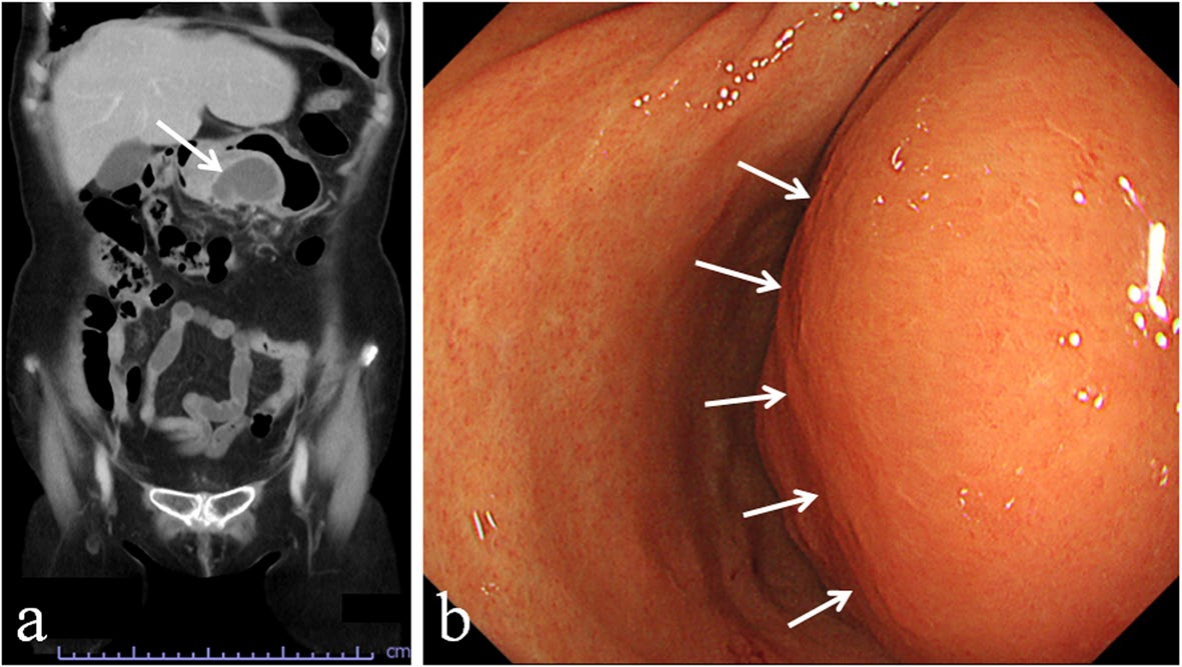
Findings of CT and upper gastrointestinal endoscopy. Coronal section of CT revealed an intramural cystic lesion of the stomach (**a**). Upper gastrointestinal endoscopy showed an elevated lesion covered with normal mucosa in the gastric antrum (**b**). Arrows in **a** and **b** indicate the lesion

A 71-year-old Japanese woman complaining of epigastric pain came to Hyogo College of Medicine Hospital. Computed tomography showed an intramural cystic tumor having a maximum diameter of 70 mm in the antrum of the stomach (Fig. 1a). Upper gastrointestinal endoscopy showed an elevated lesion covered with normal mucosa in the gastric antrum (Fig. 1b), and endoscopic ultrasound-guided fine needle aspiration (EUS-FNA) biopsy and incisional biopsy were performed for the lesion. A large amount of mucinous or pus-like material was discharged from the biopsy site. Histopathological examination of the biopsy specimen only revealed abscess-like inflammatory granulation with mucus (data not shown). Both upper gastrointestinal endoscopy and computed tomography two weeks after the biopsy showed that the lesion decreased in size but remained. There was a possibility that the lesion was intramural gastric abscess of unknown etiology. However, the patient was decided to be underwent tumor resection because it could not be ruled out that the lesion was GIST with cystic change. Laparoscopic distal gastrectomy with Roux-en Y anastomosis was done. During the procedure, gastric antral wall thickening with adhesion to the omentum and transverse mesocolon was observed, but the tumor resection was completely accomplished. Lymph node dissection was not carried out. The patient has no recurrent lesion for 8 months after the surgery.

**Fig. 2:**
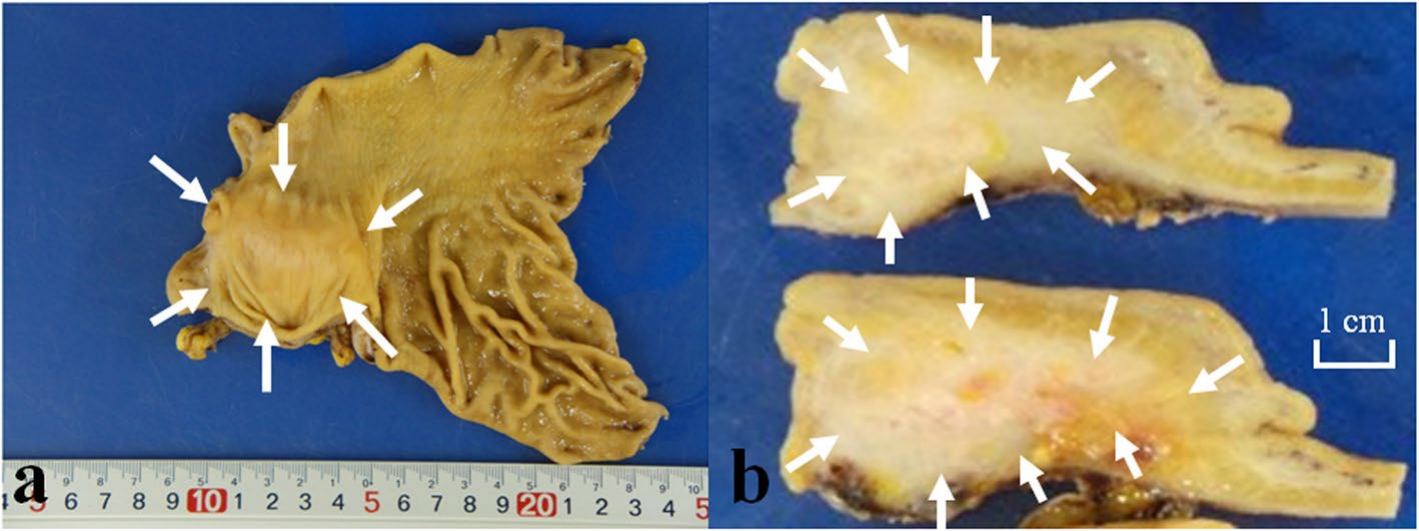
Macroscopic findings of the resected stomach and cut surface of the sliced specimens. Resected stomach showed a flat elevated lesion in the antrum (**a**). Cut surface of the specimens longitudinally sliced at the central portion of the lesion appeared to be solid but not cystic (**b**). Arrows in (**a**) and (**b**) indicate the lesion

**Fig. 3:**
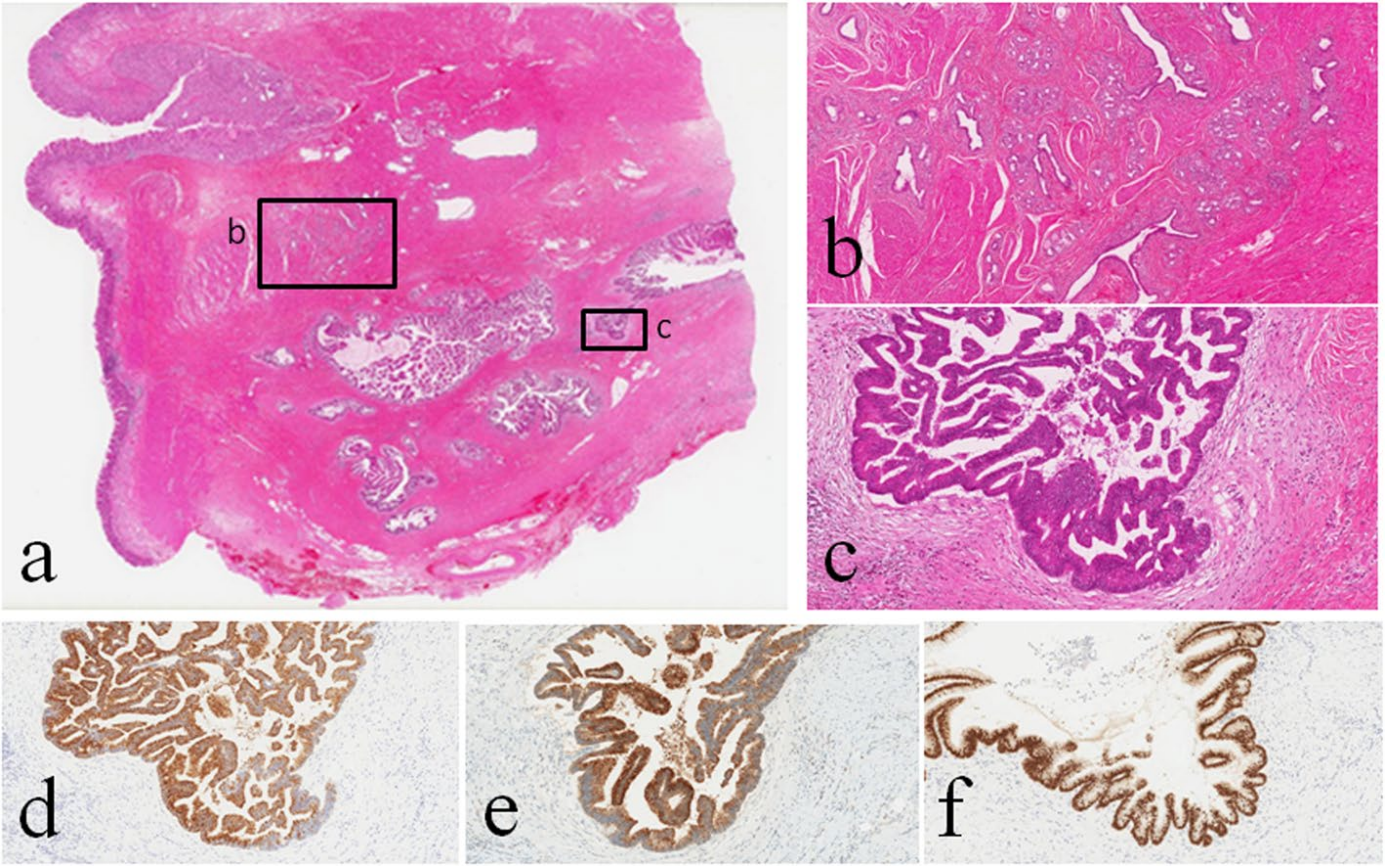
Histological and immunohistochemical findings of the gastric intramural lesion. Low and medium power views of the specimen showed that the lesion was ectopic pancreas located mainly in the proper muscular and subserosal layers (**a**, **b**). Dilated duct components with tubulo-villous epithelial proliferation at the deep portion of the lesion were consistent with pancreatic IPMN (**c**). The covering epithelial cells were MUC2- (**d**), MUC5AC- (**e**), and CDX2-positive (**f**). **b**, **c** High-power images of insets **b** and **c** in **a**

Resected tissue was fixed in 10% neutral buffered formalin. A slightly elevated lesion (45 × 40 mm) was observed in the gastric antrum (Fig. 2a). Cut surface of the lesion did not appear clearly cystic but did rather solid, and the lesion seemed to be present mainly in the proper muscular and subserosal layers (Fig. 2b). Tissues were embedded in paraffin and 3-μm-thick sections were cut. The sections were used for hematoxylin and eosin staining and IHC by detection system of BOND Polymer Refine Detection (Leica Biosystems, Wetzlar, Germany). Histology showed that the gastric intramural lesion was ectopic pancreas measuring 35 × 25 × 17 mm which was located mainly in the proper muscular and subserosal layers (Fig. 3a, b). The lesion contained all the components of ducts, acini and islets of Langerhans (Heinrich type I), although the acini and islets were rare components (data not shown). IHC showed that the acini were positive for trypsin and the islets were positive for chromogranin, synaptophysin and CD56 (data not shown). Inflammatory granulation with abscess was observed at the superficial portion of the lesion (data not shown).

**Fig. 4:**
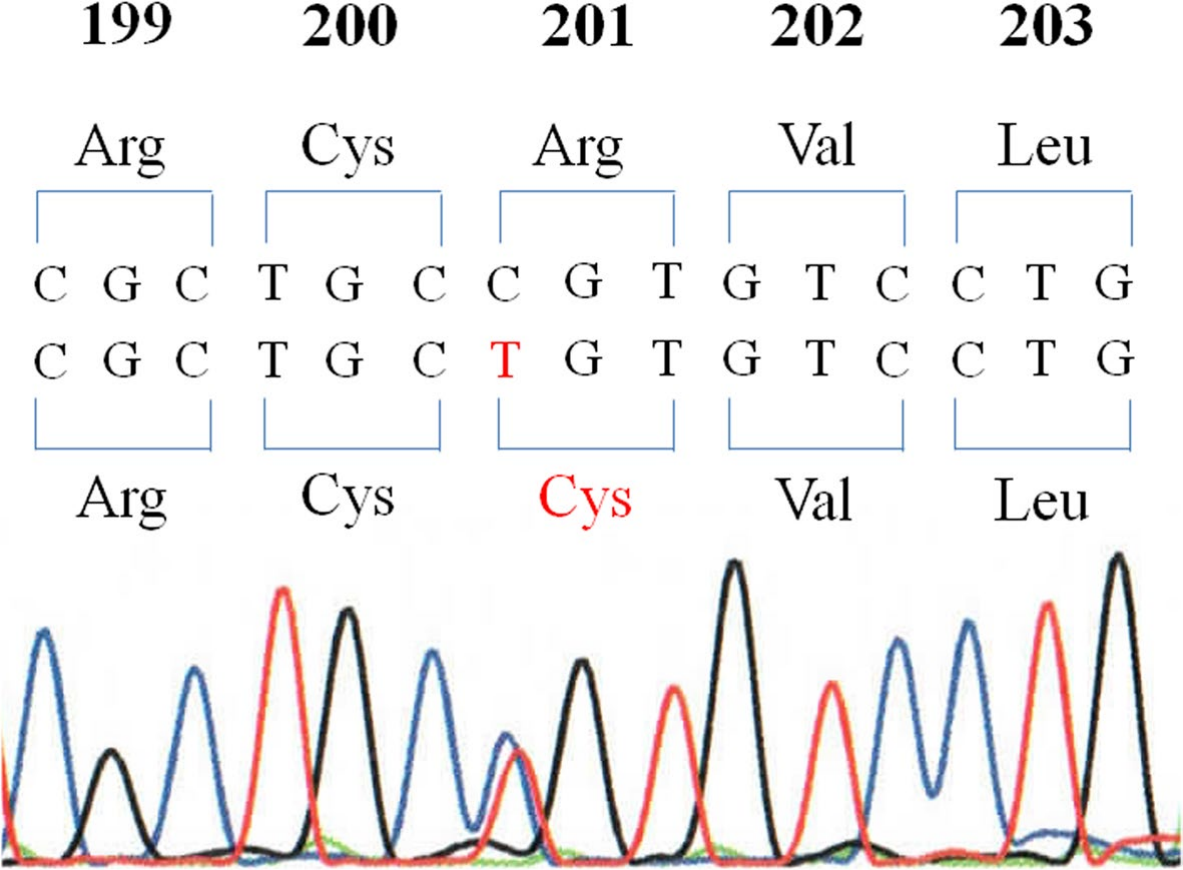
Mutational analysis of *GNAS* gene using genomic DNA extracted from histological specimen. IPMN component associated with ectopic pancreas had a heterozygous mutation of *GNAS* gene at codon 201 in exon 8 (Arg201Cys)

Moreover, the lesion contained dilated duct components with tubulo-villous epithelial proliferation (Fig. 3c) at the deep portion of the lesion consistent with pancreatic IPMN. The covering epithelial cells had atypical nuclei, and the nuclei were not diffusely but rather widely positive for TP53 by IHC (data not shown). Invasive proliferation of the tumor cells was not apparent. Fibrosis was found around and within the lesion. IHC showed that the tumor cells of IPMN component were MUC2- (Fig. 3d), MUC5AC- (Fig. 3e) and CDX2-positive (Fig. 3f) but MUC1- and MUC6-negative (data not shown). Mutational analyses using genomic DNA extracted from histological specimen revealed that the lesion had heterozygous *GNAS* mutation at codon 201 in exon 8 (Arg201Cys) (Fig. 4) but not *K-ras* mutation at exon 2 and *TP53* mutation at exon 5 (data not shown). Thus, the lesion was finally diagnosed as gastric ectopic pancreas accompanied by intestinal type IPMN with high grade dysplasia (non-invasive adenocarcinoma) possessing *GNAS* mutation (Arg201Cys). The lesion was considered to be completely resected.

## Discussion

Ectopic pancreas is generally a benign disease, but there is a possibility that neoplasms occurring in the normal pancreas such as ductal adenocarcinomas and IPMNs may develop even within the ectopic pancreas [7–22]. We experienced a rare case of gastric ectopic pancreas associated with the lesion consistent with pancreatic IPMN. Since IPMN component was adjacent to the deep portion of the ectopic pancreas, IPMN was considered to be derived from duct component of the ectopic pancreas. Seventeen cases of ectopic pancreas with IPMN have been reported to date in the English literature including 6 cases of gastric one (Table 1) [7–22]. Thus, our case is the 18th case of ectopic pancreas with IPMN and the 7th case of gastric one.

**Table 1:** Summary of ectopic pancreas cases with IPMN component

Case no.	Authors and reference	Age/gender	Clinical presentation	Anatomic site	Size (mm)	Diagnosis	Mutational analyses
1	Cates et al. [7]	73/M	Lower abdominal pain, vomiting, diarrhea	Meckel’s diverticulum	7	IPMA	Not done
2	Phillips et al. [8]	80/M	Recurrent dyspepsia, intermittent diarrhea	Stomach	60	IPMN	Not done
3	Park et al. [9]	66/M	Asymptomatic	Stomach	12	IPMN	Not done
4	Rosek et al. [10]	59/F	Upper abdominal discomfort	Duodenum	50	IPMC	Not done
5	Patel et al. [11]	44/M	Abdominal bloating, epigastric pain	Stomach	30	IPMN with low-grade dysplasia	Not done
6	Tsapralis et al. [12]	60/M	Jaundice, dark urine, pale stool	Stomach	25	IPMN	Not done
7	Song et al. [13]	74/M	Direct and rebound tenderness of the lower abdomen	Jejunum	30	Adenocarcinoma associated with IPMN	Not done
8	Okamoto et al. [14]	75/F	Asymptomatic	Jejunum	15	IPMN with low-grade dysplasia	Not done
9	Lawrence et al. [15]	47/F	Intermittent nausea, right upper quadrant discomfort	Proximal hepatic duct	Unknown	IPMN	Not done
10	Lee et al. [16]	49/F	Asymptomatic	Ileum	22	IPMN with high-grade dysplasia	Not done
11	Ma et al. [17]	74/M	Ileocecal fistula, ileal stricture	Meckel’s diverticulum	9	Incipient IPMN with focal high-grade dysplasia	*K-ras*: Gly12Asp (+)
12	Ma et al. [17]	70/M	Crohn disease, ileal stricture	Meckel diverticulum	35	IPMN with focal high-grade dysplasia	*K-ras*: Gly12Asp (+)
13	Christopher et al. [18]	Unknown/unknown	Asymptomatic	Stomach	30	Adenocarcinoma associated with IPMN	Not done
14	Noda et al. [19]	54/F	Sudden abdominal pain	Jejunum	12	IPMN	Not done
15	Addeo et al. [20]	69/M	End-stage renal disease	Stomach	60	IPMN with low-grade dysplasia	Not done
16	Hisanaka et al. [21]	70/F	Asymptomatic	Duodenum	Unknown	Adenocarcinoma with IPMN	*K-ras*: Gly12Asp (+)
17	Kim et al. [22]	46/F	Asymptomatic	Stomach	20	IPMN with low-grade dysplasia	Not done
18	Our case	71/F	epigastric pain	Stomach	35	IPMN with high-grade dysplasia	K-ras: Gly12Asp (-) GNAS: Arg201Cys (+)

Pathological changes other than neoplasms such as cyst formation and inflammation/abscess which can occur in the normal pancreas could also happen in ectopic pancreas. The lesions might cause symptoms such as abdominal pain, epigastric discomfort, nausea, vomiting, and bleeding [23], although most ectopic pancreas remains asymptomatic. In our case, the patient complained of epigastric pain. Since computed tomography revealed an intramural cystic tumor having a maximum diameter of 70 mm in the gastric antrum before biopsy and since the biopsy specimen showed abscess-like component with mucus, we retrospectively consider that cystic dilatation of IPMN lesion with concomitant abscess formation could cause her epigastric pain. Ectopic pancreas of Heinrich types I and II possessing acinar component appears to be associated with acute pancreatitis-like change due to some obstructive mechanism of the ductal component [24]. Abscess-like component observed in the biopsy and resected samples of our patient might be the case of acute pancreatitis-like change.

Pancreatic IPMN exhibits papillary epithelial proliferation and mucus production, and is classified into four subtypes (gastric, intestinal, pancreatobiliary, and oncocytic types) according to mucus traits and histological features [3, 4]. Intestinal type IPMN is a frequently observed subtype among them [3, 4]. Even in IPMN associated with ectopic pancreas, intestinal type appears to be a frequent subtype [14, 16, 19, 21]. Colonic villous adenoma-like growth pattern and immunohistochemical profile of MUC2-, MUC5AC- and CDX2-positivities but MUC1- and MUC6-negativities in the present case indicated that the lesion was consistent with the intestinal type IPMN.

Pancreatic IPMNs specifically have *GNAS* mutation at exon 8 (Arg201Cys or Arg201His) among pancreatic neoplasms [5, 6]. Approximately half of pancreatic IPMNs have *GNAS* mutation but none of pancreatic duct adenocarcinomas have such a mutation [5]. On the other hand, about 80% of pancreatic duct adenocarcinomas have *K-ras* mutation, while approximately half of pancreatic IPMNs have the mutation [5]. Thus, approximately each quarter of pancreatic IPMNs have both *GNAS* and *K-ras* mutations, only *GNAS* mutation, only *K-ras* mutation, or neither of them. Each subtype (gastric, intestinal, pancreatobiliary or oncocytic type) of pancreatic IPMNs appears to have *GNAS* and/or *K-ras* mutations at similar frequency [5]. In the present case, we detected *GNAS* mutation but not *K-ras* mutation. This is the first reported case of ectopic pancreas with IPMN component which is proved to have *GNAS* mutation. Since all of intestinal type IPMNs have GNAS mutations and since invasive cancer associated with IPMNs appears to be much more frequent in intestinal type IPMN, detection of GNAS mutation in the specimen from intramural gastric lesion by preoperative EUS-FNA biopsy/cytology could become an indicator suggesting that the tumor should be excised.

Imaging tests could suspect ectopic pancreas of the stomach, but preoperative accurate diagnosis of the lesion by them is usually difficult. In the case of gastric ectopic pancreas with IPMN, the situation may be similar. Moreover, preoperative EUS-FNA biopsy/cytology and incisional biopsy cannot always obtain appropriate material for pathological diagnosis like in our case. Thus, definitive diagnosis of gastric ectopic pancreas without IPMN or with IPMN is often made by the histological examination of the resected tissue. Many gastric GISTs have solid appearance by imaging, but some GISTs might show cystic change especially in those with platelet-derived growth factor receptor alpha gene mutation. Indeed, preoperative clinical diagnosis of our case was GIST with cystic change, and the final diagnosis of ectopic pancreas with IPMN was made by the resected specimen. Although both ectopic pancreas with IPMN and GIST with cystic change have to be resected because of possibility with malignant characteristics, systematic lymph node dissection might not be required in the case of GISTs because they usually could not metastasize to lymph node. In any case, we have to consider that gastric intramural lesion with cystic change might be ectopic pancreas with IPMN component.

## Conclusions

We experienced an extremely rare case of gastric ectopic pancreas accompanied by intestinal type IPMN with high grade dysplasia possessing *GNAS* mutation. This is the first case of ectopic pancreas with IPMN which was proved to have *GNAS* mutation. Intimate preoperative examinations including imaging analyses and EUS-FNA biopsy/cytology are recommended to decide whether the lesion should be resected or not even if they are not effective for getting the accurate diagnosis.

## Data Availability

All data supporting the findings of this study are available within the article.

## Abbreviations

IPMNs: Intraductal papillary mucinous neoplasms
EUS-FNA: Endoscopic ultrasound-guided fine needle aspiration
IHC: Immunohistochemistry
MUC: Mucin core protein
GIST: Gastrointestinal stromal tumor
